# An analog-AI chip for energy-efficient speech recognition and transcription

**DOI:** 10.1038/s41586-023-06337-5

**Published:** 2023-08-23

**Authors:** S. Ambrogio, P. Narayanan, A. Okazaki, A. Fasoli, C. Mackin, K. Hosokawa, A. Nomura, T. Yasuda, A. Chen, A. Friz, M. Ishii, J. Luquin, Y. Kohda, N. Saulnier, K. Brew, S. Choi, I. Ok, T. Philip, V. Chan, C. Silvestre, I. Ahsan, V. Narayanan, H. Tsai, G. W. Burr

**Affiliations:** 1 IBM Research – Almaden, San Jose, CA USA; 2 IBM Research – Tokyo, Kawasaki, Japan; 3 IBM Research – Albany NanoTech Center, Albany, NY USA; 4 IBM Thomas J. Watson Research Center, Yorktown Heights, NY USA

**Keywords:** Electrical and electronic engineering, Electronic devices, Information technology, Computational science

## Abstract

Models of artificial intelligence (AI) that have billions of parameters can achieve high accuracy across a range of tasks^1,2^, but they exacerbate the poor energy efficiency of conventional general-purpose processors, such as graphics processing units or central processing units. Analog in-memory computing (analog-AI)^3–7^ can provide better energy efficiency by performing matrix–vector multiplications in parallel on ‘memory tiles’. However, analog-AI has yet to demonstrate software-equivalent (SW_eq_) accuracy on models that require many such tiles and efficient communication of neural-network activations between the tiles. Here we present an analog-AI chip that combines 35 million phase-change memory devices across 34 tiles, massively parallel inter-tile communication and analog, low-power peripheral circuitry that can achieve up to 12.4 tera-operations per second per watt (TOPS/W) chip-sustained performance. We demonstrate fully end-to-end SW_eq_ accuracy for a small keyword-spotting network and near-SW_eq_ accuracy on the much larger MLPerf^8^ recurrent neural-network transducer (RNNT), with more than 45 million weights mapped onto more than 140 million phase-change memory devices across five chips.

## Abstract (web summary)

A low-power chip that runs AI models using analog rather than digital computation shows comparable accuracy on speech-recognition tasks but is more than 14 times as energy efficient.

## Main

**Fig. 1:**
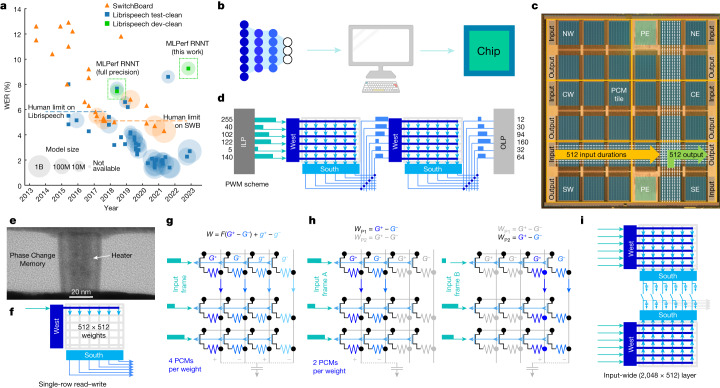
Chip architecture. **a**, Speech recognition has improved markedly over the past 10 years, driving down the WER for both the Librispeech and SwitchBoard (SWB) datasets, thanks to substantial increases in model size and improved networks, such as RNNT or transformer. For comparison with our results, the MLPerf RNNT full-precision WER is shown for two Librispeech datasets (‘test-clean’ and ‘dev-clean’)^8^, along with this work’s WER, which was computed on Librispeech dev-clean. For model size: B, 1 billion; M, 1 million. **b**, Inference models are trained using popular frameworks such as PyTorch or TensorFlow. Further optimization for analog AI can be achieved with the IBM analog HW acceleration kit (https://aihwkit.readthedocs.io/en/latest/). **c**, Trained model weights are then used on a 14-nm chip with 34 analog tiles, two processing elements (PE, not used for this work) and six ILP–OLP pairs. Tiles are labelled as north (N), centre (C) or south (S) followed by west (W) or east (E). **d**, Each ILP converts 512 8-bit inputs into 512 element vectors of pulse-modulated durations, which are then routed to the analog tiles for integration using a fully parallel 2D mesh that allows multi-casting to multiple tiles. After MAC, the charge on the peripheral capacitors is converted into durations^4^ and sent either to other tiles, leading to new MACs, or to the OLP, where durations are reconverted into 8-bit representations for off-chip data-processing. **e**, Transmission Electron Microscopy (TEM) image of one PCM. **f,** Each tile contains a crossbar array with 512 × 2,048 PCMs, programmed using a parallel row-wise algorithm^4^. **g**, PCMs can be organized in a 4-PCM-per-weight configuration, with G^+^, g^+^ adding and G^−^, g^−^ subtracting charge from the peripheral capacitor, with a significance factor *F* (which is 1 in this paper). **h**, Alternatively, they can have a 2-PCM-per-weight configuration, which achieves a higher density. By reading different input frames through weights *W*_P1_ or *W*_P2_, a single tile can map 1,024 × 512 weight layers. **i**, Finally, two adjacent tiles can share their banks of 512 peripheral capacitors, enabling integration in the analog domain across 2,048 input rows.

The past decade has seen AI techniques spread to a wide range of application areas, from the recognition and classification of images and videos^9^ to the transcription and generation of speech and text^10–16^, all driven by a relentless progression towards deep neural network (DNN) models with ever more parameters. In particular, transformer^1^ and recurrent neural-network transducer (RNNT)^12,13,16^ models containing up to one billion parameters^2^ have produced a marked decrease in word error rate (WER) (and therefore much better accuracy) for the automated transcription of spoken English-language sentences, as shown in Fig. 1a for two widely used datasets, Librispeech^17^ and SwitchBoard^18^. Unfortunately, hardware (HW) performance has not kept pace, leading to longer training and inference times and greater energy consumption^19^. Large networks are still trained and implemented using general-purpose processors such as graphics processing units and central processing units, leading to excessive energy consumption when vast amounts of data must move between memory and processor, a problem known as the von Neumann bottleneck.

Analog-AI HW avoids these inefficiencies by leveraging arrays of non-volatile memory (NVM) to perform the ‘multiply and accumulate computation’ (MAC) operations which dominate these workloads directly in the memory^3–7^. By moving only neuron-excitation data to the location of the weight data, where the computation is then performed, this technology has the potential to reduce both the time and the energy required. These advantages are further enhanced for DNN models that have many large fully connected (FC) layers, such as the RNNT or transformer models used for state-of-the-art natural language processing (NLP). In conventional digital implementation, such layers require enormous movement of data but provide scant opportunity for amortization over subsequent computing. For analog AI, by contrast, such layers are efficiently mapped onto analog crossbar arrays and computed in parallel using a single integration step. Given the finite endurance and the slow, power-hungry programming of NVM devices, such analog-AI systems must be fully weight stationary, meaning that every weight must be preprogrammed before inference workload execution begins.

A highly heterogeneous and programmable accelerator architecture for analog AI has been introduced^20^ for which system-level performance assessments have predicted energy efficiencies 40–140 times higher than those of cutting-edge graphics processing units. However, this simulation study required several design assumptions that have yet to be demonstrated in HW, two of which are directly addressed below. The first is the use of a dense and efficient circuit-switched 2D mesh to exchange massively parallel vectors of neuron-activation data over short distances. The second is the successful implementation of DNN models that are large enough to be relevant for commercial use and are demonstrated at sufficiently high accuracy levels.

In this paper, we present experimental results using a 14-nm inference chip leveraging 34 large arrays of phase-change memory (PCM) devices^4^, digital to analog input, analog peripheral circuitry, analog to digital output and massively parallel-2D-mesh routing. Our chip does not include on-chip digital computing cores or static random access memory (SRAM) to support the auxiliary operations (and data staging) needed in an eventual, marketable product. However, we can use it to demonstrate the accuracy, performance and energy efficiency of analog AI on NLP inference tasks, either by implementing simple operations such as rectified linear unit (ReLU) non-linear function directly in the analog domain or by performing small amounts of auxiliary computing off-chip.

To demonstrate the flexibility of the chip, we chose two neural-network models from the MLPerf standard benchmark^8^, a suite of industry-relevant use cases. We first targeted the tiny-model task of keyword-spotting network (KWS) on the Google speech-commands dataset. For this we used a HW-aware (HWA) trained network, retrained using a variety of techniques available in the open-source IBM analog HW acceleration kit (https://aihwkit.readthedocs.io/en/latest/) (Fig. 1b). We then implemented the MLPerf version of RNNT, a large data-center network, on Librispeech without any additional HWA retraining. This model has 45 million weights, which we implement using more than 140 million PCM devices across five packaged chip modules, demonstrating near-SW_eq_ accuracy (ours is 98.1% of that exhibited by the base software (SW)-only model) and executing about 99% of the operations on the analog-AI tiles.

## Chip architecture

A micrograph of the chip is shown in Fig. 1c, highlighting the 2D grid of 34 analog tiles, each of which has its own 512 × 2,048 PCM crossbar array. Tiles are grouped into six power domains, labelled as north, centre or south followed by west or east. Each power domain contains one input landing pad (ILP) (Fig. 1d) and one output landing pad (OLP), each associated with a large SRAM. The ILP receives digital input vectors (each vector has 8-bits unsigned integer (UINT8) × 512 entries) from off-chip, converting these inputs into pulse-width-modulated (PWM) durations onto 512 wires situated in parallel at the edge of the 2D mesh running over all the tiles^4,20^. Conversely, the OLP receives PWM durations on 512 wires, digitizing these durations back into UINT8 for off-chip data transport.

Analog-tile to analog-tile communication is performed using durations, eliminating the area, power and latency associated with analog-to-digital conversion at the tile periphery^4^ for situations in which integration on the rows of each destination tile can be performed synchronously with the readout of the columns of one or more source tiles, including FC layers with simple activation functions. When duration vectors are sent from a tile to the OLP, the chip is effectively implementing a ramp-based analog-to-digital converter (ADC), except that the shared ramp circuits and dedicated comparators are located at the tiles and the digital counters are at the OLP. Digitization becomes a necessity for transformer attention and models that require internal data staging.

PCM devices are integrated in the back-end wiring above 14-nm front-end circuitry (Fig. 1e) and can encode analog conductance states by tuning, with electrical pulses, the relative volume of crystalline-phase (highly conductive) and amorphous-phase (highly resistive) material at the narrow bottom electrode. To program PCM devices, a parallel programming scheme is used (Fig. 1f) so that all 512 weights in the same row are updated at the same time^4^.

Weights can be encoded using a variable number of PCM devices. Figure 1g shows a 4-PCM-per-weight configuration, where each of the four PCM devices contributes equally to the read current and thus to the charge stored on the peripheral capacitor. A second, denser scheme uses a 2-PCM-per-weight set-up (Fig. 1h), encoding one weight, *W*_P1_ = *G*^+^ - *G*^−^, on the first two PCM devices and a different weight, *W*_P2_ = *g*^+^ - *g*^−^, on the second pair of devices. In this way, two different input vectors can be multiplied with *W*_P1_ and *W*_P2_ in two separate time steps, on the same capacitor, allowing analog MAC across 1,024 rows. Finally, two analog tiles can share one bank of peripheral capacitors (Fig. 1i), further extending the analog integration up to 2,048 analog input rows across 512 columns per pair of tiles.

**Fig. 2:**
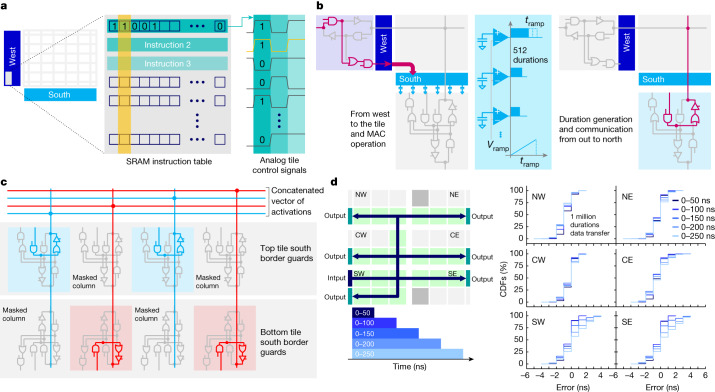
Reconfigurable architecture and routing. **a**, For maximum test-time flexibility, each tile contains a user-programmable LC that defines all timing sequences. **b**, LC controls signal routing through the 2D mesh from ILP to the tile, MAC, output duration generation through ramp plus comparator circuitry and borderguard tile routing configuration. **c**, As an example, LC can implement 2D-mesh concatenation such as merging the durations originating from the even columns on one tile with the durations coming from the odd columns of another tile. **d**, To test the communication, 1 million random input durations are multi-cast, in parallel, to all 6 OLPs. Durations randomly vary between 0 and 50 ns (dark-blue lines) or between 0 and either 100, 150, 200 or 250 ns (lighter shades of blue) with 1-ns granularity. Cumulative distribution functions (CDFs) reveal that the communication error never exceeds 5 ns, demonstrating high transport accuracy.

All weight configurations, MAC operations and routing schemes are defined with a user-configurable local controller (LC) available on each tile (Fig. 2a). A local SRAM stores all the instructions defining the time sequence of several-hundred control signals, allowing for a highly flexible test and simplifying design verification, with a small area penalty when compared with predefined-state machines.

The 2D mesh comprises 512 east–west wires and 512 north–south wires sitting over each tile, with a diagonal set of 512 metal vias to connect each corresponding pair of wires. ‘Borderguard’ circuits at the four edges of each tile can block or propagate each duration signal using tri-state buffers, mask bits and digital logic. This allows complex routing patterns to be established and changed when required by the LC, including a multi-cast of vectors to multiple destination tiles, and a concatenation of sub-vectors originating from different source tiles^20^ (Fig. 2c). Finally, Fig. 2d verifies that durations can be reliably transmitted across the entire chip, with a maximum error equal to 5 ns (3 ns for shorter durations).

## KWS task

**Fig. 3:**
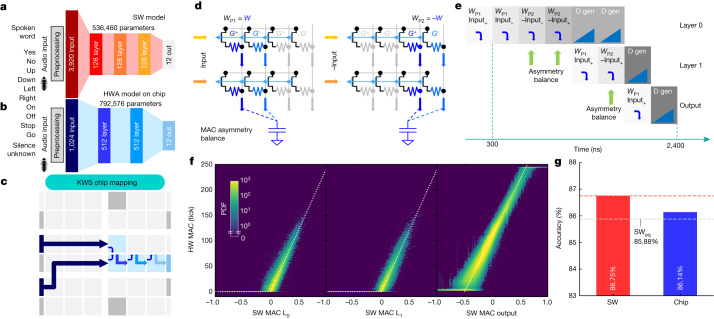
End-to-end KWS task. **a**, To classify spoken words into one of the 12 highlighted classes for KWS, an FC baseline is used as a reference. **b**, The network is then retrained using HWA techniques. **c**, The end-to-end implementation uses four analog tiles. **d**, An AB method is used to increase MAC accuracy. Weights *W* and −*W* are programmed on *W*_P1_ and *W*_P2_ respectively. By inferencing the desired input *x* on *W*_P1_ and then −*x* on *W*_P2_, the MAC is collected twice (*xw* + (−*x*) × (−*w*)), cancelling out any fixed peripheral circuitry asymmetries and improving MAC accuracy. **e**, A timing diagram shows that a full frame is processed in 2.4 μs. Because the ReLU activation (implemented on-chip in the analog domain) generates positive-only outputs, the second layer requires only two integration steps, rather than the four needed in the first layer. **f**, Experimental activations after layers L_0_, L_1_ and output correlate closely with ideal SW MACs calculated using HW input. PDF, probability distribution function. **g**, This leads to SW_eq_ accuracy for this fully end-to-end demonstration.

To demonstrate the performance of the chip in an end-to-end network, we implemented a multi-class KWS task^21^. MLPerf classifies KWS as a ‘tiny’ inference model^8^ and proposes a convolutional-neural-network architecture trained on the Google Speech Commands dataset comprising 12 keywords (Fig. 3a). For this implementation, we instead adopted an FC network^22^. Both networks require upstream digital preprocessing to convert incoming audio waveforms into suitable input data vectors using a feature-extraction algorithm^21,22^. The FC model achieves a classification accuracy of 86.75%, compared with 90% for the MLPerf convolutional neural network, but offers a simpler architecture and faster performance (several KWS open submissions to MLPerf use FC-type networks, sometimes reporting even lower accuracy around 82.5%)^8^. Because an FC network matches our chip topology and exploits our large tiles, our goal is to match the available SW accuracy of 86.75%.

To enable a fully end-to-end implementation on our chip, we first modified the audio-spectrum digital preprocessing to produce 1,960 inputs and increased the size of each hidden layer from 128 to 512 for our tiles (in 4-PCM-per-weight mode). To make the network more resilient to analog noise^23–26^, we retrained it while including weight and activation noise, weight clipping, L2 regularization and bias removal (https://aihwkit.readthedocs.io/en/latest/). We then pruned this trained network down to 1,024 inputs (Fig. 3b) to fit the first layer into a two-tile mapping configuration (Fig. 3c), using the shared-capacitor-bank approach shown in Fig. 1i. Our end-to-end implementation uses four tiles in total: two for the first weight layer and two for the next two weight layers.

To improve the MAC accuracy and compensate for asymmetries in the peripheral circuits, we introduce a MAC asymmetry balance (AB) method (Fig. 3d). Actual weights, *W*, are programmed on the first PCM pair, *W*_P1_, and opposite-signed weights, −*W*, are encoded on the second PCM pair, *W*_P2_. By first multiplying the actual input on *W*_P1_ = *W* and then −input on *W*_P2_ = −*W*, we computed the desired MAC (scaled by ×2) while cancelling out fixed asymmetries in the peripheral circuitry for current collection.

Each audio frame requires 2.4 μs in total, in the form of 8 time steps of 300 ns each (Fig. 3e); this is 7 times faster than the best-case latency currently reported by MLPerf^8^. Experimentally measured MAC-plus-Activation function (ReLU for layers L_0_ and L_1_, linear for Output) correlations with the expected SW result are shown in Fig. 3f for all three layers. The measured KWS accuracy is 86.14% (Fig. 3g), well within the MLPerf SW_eq_ ‘iso-accuracy’ limit of 85.88% (defined as 99% of the accuracy of the original SW model).

## RNNT

Although KWS represents an excellent benchmark for very small models, we can also use our chip to demonstrate much larger and more-complex networks. As an example, the NLP task of speech-to-text transcription enables applications such as agent assist, media content search, media subtitling, clinical documentation and dictation tools (https://aws.amazon.com/what-is/speech-to-text/). We therefore implemented the MLPerf Datacenter network RNNT as an industry-relevant workload demonstration. To further simplify model use, we programmed the MLPerf weights directly with no additional HWA retraining.

**Fig. 4:**
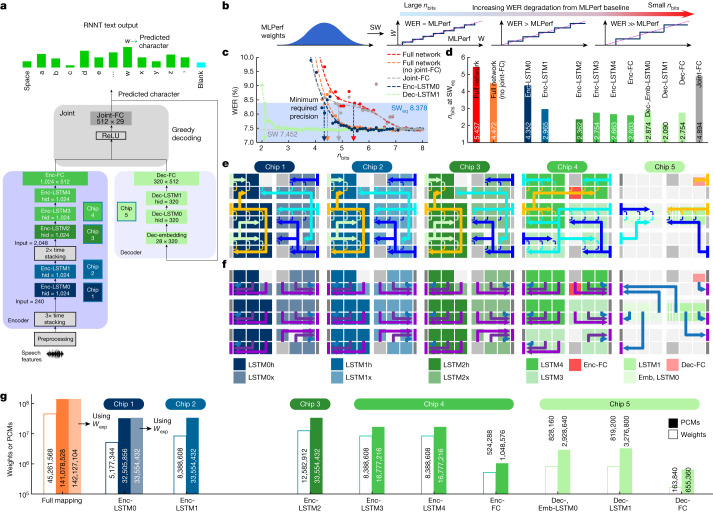
MLPerf RNNT network for speech transcription. **a**, MLPerf RNNT model, trained on the Librispeech dataset, comprises encoder (Enc), decoder (Dec) and joint blocks. The input signal is digitally preprocessed and stacked to generate the input of Enc-LSTM0 (chip 1) and Enc-LSTM1 (chip 2). The resulting output vectors are again time-stacked before feeding a 2,048-input Enc-LSTM2 (chip 3), followed by two 1,024-input Enc-LSTM3,4 and an Enc-FC linear layer (chip 4). The resulting encoder output is then merged with the vectors received from the Dec (chip 5). Finally, a joint-FC calculates the next-letter probability (in SW), which feeds back to the Dec. This entails greedy decoding in which the highest probability selects the output letter. **b**, SW-based sensitivity analysis performed by progressively quantizing the FP32 MLPerf weights. **c**, The WER increases beyond the SW_eq_ limit when weights are excessively quantized. **d**, There is a threshold *n*_bits_ at which the WER is still SW_eq_ for the full network, the full network without joint-FC quantization, and for each individual layer. While Dec-LSTM1 is the most resilient to noise, joint-FC exhibits significant sensitivity and is small in size, so it is not mapped in analog to preserve high accuracy. **e**, All the other layers are mapped to analog tiles (mapping details in Extended Data Figs. 5, 7). All arrows show the input signal routing and are operating at the same time, each performing a simultaneous multi-cast to all tiles that show the same-colour MAC arrow. Note that the borderguard circuits can enable duration data arriving at the west side of a tile to deliver durations onto the rows of that tile, and a completely different duration-vector passes over the centre of that tile on its routing wires at the same time. Small arrows indicate how MACs are aggregated in the analog domain across tile pairs. **f**, The output duration routing. Each arrow colour requires its own time slot: three for chips 1, 2, 3 and 4, and one for chip 5. Output routing from tiles to OLPs can involve implicit concatenation (chip 5). More details are given in the Methods. The joint block and all LSTM vector–vector operations are computed off-chip. **g**, More than 45 million weights are mapped using more than 140 million PCMs, with an average of 2.9 (3.1 with *W*_exp_) PCMs per weight. Coloured bars show PCMs, white bars show weights.

The MLPerf RNNT showcases all the important building blocks, such as a multilayer encoder (Enc), decoder (Dec) and joint subnetwork blocks (Fig. 4a). The network is slightly simplified with respect to state-of-the-art RNNTs; the long short-term memory (LSTM) blocks are unidirectional, rather than bidirectional, and the decoding scheme is greedy rather than beam-search, which increases the WER slightly but makes online continuous-streaming use much more straightforward^27^.

### RNNT mapping on chip

As with KWS, digital preprocessing first converts raw audio queries into a sequence of suitable input data vectors. At each sequence time step, the encoder cascades data vectors through five successive LSTMs (Enc-LSTM0, 1, 2, 3, 4) and one FC layer (Enc-FC). At each LSTM, the local input vector for that layer is concatenated with a local ‘hidden’ vector, followed by vector–matrix multiplication through a very large FC weight layer, producing four intermediate sub-vectors. These sub-vectors are then processed and combined using a relatively small amount of vector–vector computing, generating an output vector that is sent forward to become the input to the next LSTM or FC layer for that same time step, and also recursively fed back to become its own hidden vector for the next time step. Time-stacking, performed immediately after preprocessing, as well as between Enc-LSTM1 and Enc-LSTM2 (Fig. 4), scales down the effective number of time steps in the local sequence by concatenating multiple arriving data vectors into one departing data vector.

The Dec block, which operates in parallel with the encoder, consists of one embedding FC layer (Dec-Emb), two LSTMs (Dec-LSTM0, 1) and one FC layer (Dec-FC). Finally, the joint layer sums the Enc and Dec signals, applies a ReLU activation function and selects the predicted output letter (including the possibility of a ‘blank’ character) for that time step using a 512 × 29 FC layer with a greedy decoding scheme. The predicted output letter is both the model output and the next input to the Dec block. The joint block alternates between emitting blanks, at which point the next encoder output is consumed, and emitting letters, which then triggers Dec processing. As a result, the number of Dec iterations will not usually match the input sequence length seen by the encoder.

When large DNNs such as RNNT are implemented with reduced digital precision, optimal precision choices may vary across the network^28–30^. Similarly, implementation in analog-AI HW also requires careful layer-specific choices to balance accuracy and performance. Although dense 2-PCM-per-weight mapping (Fig. 1h) can improve energy efficiency (increasing the number of operations per second per watt, OPS/W) or areal efficiency (the number of operations per mm^2^), higher accuracy can be achieved using techniques such as AB, in exchange for increased area, energy and/or time. Therefore, before mapping RNNT on HW, we need to find out which network layers are particularly sensitive to the presence of weight errors and other analog noise.

We perform this initial assessment in SW, not by adding random noise (on either weights or activations) and repeating ad nauseam to obtain stable results through Monte Carlo sampling, but by introducing increasingly stronger weight quantization on the whole, or just a portion, of the RNNT network (Fig. 4b). Any parts of the network outside the portion being stress-tested are evaluated using the original 32-bit floating point (FP32) precision. The resulting degradation in WER can be plotted as a function of the effective precision, *n*_bits_. Layers or entire network blocks that are less susceptible will still deliver a low WER even with aggressive quantization (small values of *n*_bits_), whereas highly sensitive blocks will exhibit a high WER even for small amounts of weight quantization.

Figure 4c shows this simulated WER as a function of *n*_bits_ for various cases, using the 99% SW_eq_ limit (an 8.378% WER) of the network baseline (7.452% WER) to identify a threshold *n*_bits_ (arrows). When weights across the full network are all quantized, WER is no longer SW_eq_ once *n*_bits_ < 5.4 (42 levels).

Repeating this process for each individual layer identifies the most-sensitive layers (those exhibiting a higher *n*_bits_ threshold (Fig. 4d)), such as the joint-FC and Enc-LSTM0, followed by Enc-LSTM1. Given the small size (512 × 29 weights) but large WER impact of the joint-FC, we chose to implement this layer within the digital processing. Again, because the chip does not contain any explicit digital processing, this joint-FC, all vector–vector products and the activation functions are computed off-chip on a host machine. The OLPs (and ILPs) are used to send data from the chip(s) to the host (and back).

Now that we have identified which layers are most sensitive, we are ready to map the MLPerf weights onto 142 tiles distributed across 5 chips. Because Enc-LSTM0 and Enc-LSTM1 are sensitive to noise, the AB method is used on these layers, together with a careful treatment of the first matrix, *W*_**x**_, of Enc-LSTM0, which helps to improve MAC accuracy and decrease WER (see Methods for details). In summary, of a total of 45,321,309 network weight and bias parameters, 45,261,568 are mapped into analog memory (99.9% of the weights). A single chip can hold only 17,825,792 weights in a 2-PCMs-per-weight scheme, so we used 5 different chips. Specific mapping details are shown in Fig. 4e,f. Coloured tiles encode weights and perform MAC operations; grey tiles are unused.

Figure 4e shows how input data reach each tile from an ILP, with fully parallel routing. After all the necessary integrations, duration vectors representing MAC results are sent from tiles to OLPs as shown in Fig. 4f. In total, more than 45 million weights are encoded using more than 140 million PCM devices, with an average of around 3 PCM devices for each weight (Fig. 4g).

### Accuracy results

**Fig. 5:**
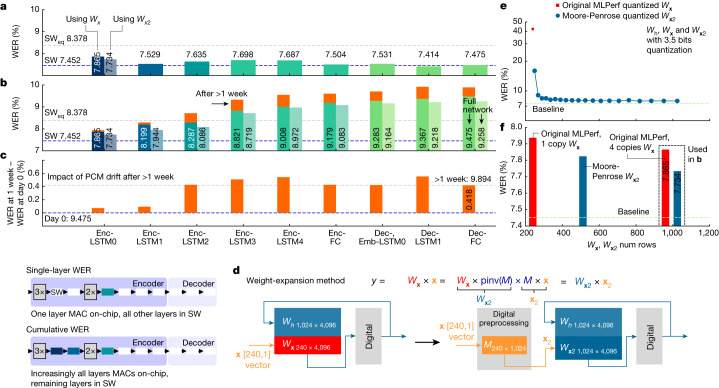
Experimental WER using Librispeech on MLPerf RNNT. **a**, Single-layer WER. The graph shows an experimental sensitivity analysis obtained by implementing one layer on-chip and all the others in SW at FP32 precision. The most critical layer is Enc-LSTM0. **b**, Cumulative WER. Full RNNT inference using all five chips on the full Librispeech validation dataset. The bars from left to right show the cumulative WER obtained when implementing increasingly more layers on-chip. The full RNNT WER, using the original MLPerf weights, achieved across five chips (right-most bar) is 9.475%. **c**, After one week of PCM drift, the cumulative WER slightly increases to 9.894%, just 0.4% more than day-0 WER. **d**, To further improve the accuracy, a weight-expansion technique is introduced for Enc-LSTM0. Given a MAC *W*_**x**_ × **x**, the insertion of a random normal matrix *M* and its pseudoinverse pinv(*M*) leads to the same MAC output. However, now *W*_**x**2_ = *W*_**x**_ × pinv(*M*) contains more rows *N*, with an increased signal-to-noise ratio. Whereas signal increases linearly with *N*, the aggregate noise across the larger number of rows increases sub-linearly ($\propto \sqrt{N}$ if noise sources are independent Gaussians). **e**, Simulation results. When quantizing Enc-LSTM0 to *n*_bits_ = 3.5 bits, the WER is 42%. Weight expansion greatly improves the resilience, even for only slightly expanded *W*_**x**2_ matrices, with the WER reduced to 7.9%, well below SW_eq_. **f**, Similar accuracy benefits are observed experimentally when implementing weight expansion on Enc-LSTM0 on-chip, revealing stronger WER reduction with respect to weight averaging. *M* × **x** is digitally preprocessed. *W*_**x**2_ expansion to 1,024 rows enables a 9.258% WER on the full RNNT, 1.81% from the SW baseline, 0.88% from SW_eq_.

Figure 5a shows the experimental WER after weight mapping and programming for the full Librispeech validation dataset of 2,513 audio queries. Here a single layer of the RNNT network is mapped on a chip, and everything else is calculated in SW. It is worth noting that individual layers of the network are SW_eq_ by themselves. As predicted in Fig. 4d, Enc-LSTM0 shows the largest WER, with other layers being more resilient to noise. Finally, the full inference experiment on all five chips is shown in Fig. 5b. From left to right, each bar reports the overall WER obtained by implementing increasingly more layers on chip. The total WER is given by the last Dec bar, 9.475%, with an overall degradation of 2.02% from the 7.452% SW baseline. For this experiment, we inference the full Librispeech validation dataset through chip 1 and save the output results. These are then input into chip 2, and so on across all 5 chips. Even when repeated after more than 1 week of PCM drift^31^,without any recalibration or weight reprogramming, the RNNT WER has degraded by only 0.4% (Fig. 5c).

We observe that the layer-to-layer WER degradation in Fig. 5b is steeper than expected from simple aggregation of the single-layer WER degradations (Fig. 5a). Intuitively, Enc-LSTM0 and other early layers have a bigger cumulative impact owing to error propagation. We can further improve the WER of Enc-LSTM0 with a new weight-expansion method involving a fixed matrix *M* with normal random values, and its Moore-Penrose pseudo-inverse, pinv(*M*) (Fig. 5d). The resultant noise-averaging helps to improve the accuracy of the MAC operation and the overall resilience of the network layer, with no additional retraining required. On analog HW, as long as the number of tiles remains unchanged, the additional cost of using more or even all of the rows in each tile is almost negligible. However, more preprocessing is needed to implement *M* × **x** in digital, although it is much less than if the entire Enc-LSTM0 layer were implemented in digital.

Using our SW-based assessment method from Fig. 4c,d, Fig. 5e shows that quantizing the Enc-LSTM0 weights to 3.5 bits leads to an excessive WER (42%). However, after weight expansion, the WER greatly decreases, even for a small *W*_**x**2_ expansion, saturating at a SW_eq_ value of 7.9% WER when *W*_**x**2_ contains 1,024 rows. The same behaviour is observed in experiments (Fig. 5f), with the WER for on-chip Enc-LSTM0 decreasing as weight expansion is increased up to a *W*_**x**2_ containing 1,024 rows, exceeding the improvement shown by simply programming multiple weight copies. Figure 5b shows that when the entire RNNT network is run on five chips, starting with expanded *W*_***x***2_ on Enc-LSTM0, WER improves to 9.258%, which is 1.81% from the SW baseline, and only 0.88% from the SW_eq_ threshold.

### Power and system performance

**Fig. 6:**
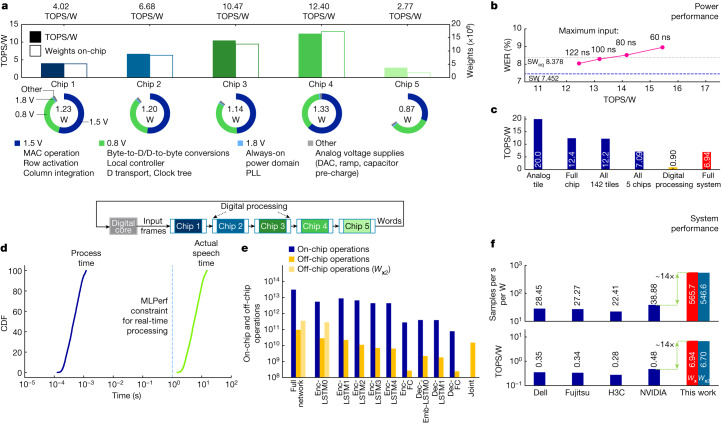
MLPerf RNNT power and system performance. **a**, Measured power and TOPS/W are shown for each chip. TOPS/W (coloured bars) correlate with the number of weights used on each chip (white bars). D, duration; DAC, digital-to-analog converter; PLL, phase-locked loop. **b**, Reducing the maximum input duration leads to an improvement in TOPS/W with only a small amount of WER degradation (chip 4 is measured, other layers in SW at FP32). **c**, Energy efficiency at various levels: analog integration only (1.5 V power domain), full chip, all 5 chips for RNNT (analog integration only and full chip), and full system level including estimated digital processing energy^20^. **d**, Simulated performance for an integrated system shows that the average processing time for each sample is 500 μs, more than 10^4^ times faster than the input speech sentence, thus enabling real-time transcription. Total processing time = 1.29 s and total real audio = 4 h 20 min, so the real-time factor ≈ 8 × 10^−5^ ≪ 1. **e**, Number of operations performed on-chip versus off-chip in the RNNT experiment, with a 325:1 ratio for the original MLPerf weights (*W*_**x**_) and 88:1 with weight expansion (W_**x**2_) (Fig. 5d). **f**, Samples per second per watt and TOPS/W performance for comparison with MLPerf submissions, showing a 14-fold improvement for our system.

We also measured the full power consumption for every chip during inference operations. The chip has various power supplies. It uses 1.5 V to drive the row activation and column integration on the tiles during analog computation. All control and communication circuits, including ILP, OLP, LC and 2D mesh, are driven at 0.8 V. As shown in Fig. 6a, the 1.5 V and 0.8 V supplies dominate power consumption. By contrast, the 1.8 V supply that drives the clock phase-locked loop (PLL) and the off-chip drivers and receivers, and some other analog voltage sources, have a negligible impact. The corresponding sustained TOPS/W values are reported in Fig. 6a. Chip 4 has the best power performance (12.40 TOPS/W) because it has the most on-chip weights. In general, the reported TOPS/W values correlate well with the number of weights encoded on-chip: chips 1 and 2 use an AB technique and have 4 PCMs per weight, whereas chip 4 uses a denser mapping of 2 PCMs per weight. Finally, the Dec chip, chip 5, has the lowest TOPS/W value because this chip implements only around 1.8 million weights across only 13 of the 34 tiles, yet the data communication is still extensive, requiring a large number of tiles and ILPs/OLPs to be active to implement the routing network (Fig. 4e,f).

Figure 6b shows that another 25% improvement in TOPS/W (from 12.4 to 15.4 TOPS/W) for chip 4 can be obtained by halving the integration time, albeit with an additional 1% degradation in the WER. Figure 6c shows how the costs of data communication, incomplete tile usage and inefficient digital computing bring the large peak TOPS/W of the analog tile itself (20.0 TOPS/W) down to the final sustained value of 6.94 TOPS/W. Given the actual chip processing times (1.5 μs for chip 5 and 2.1 μs for the other four; see Methods), we can estimate the full processing time for an overall analog–digital system (Fig. 6d). This includes the estimated computation time (and energy) if on-chip digital computing were added at the physical locations of the OLP–ILP pairs. Given the 500-μs average processing time for each audio query, the real-time factor (the ratio between processing and real audio time) is only 8 × 10^−5^, well below the MLPerf real-time constraint of 1. Although the digital compute is inefficient, the enormous ratio between the number of analog and digital operations (Fig. 6e; 325-fold for conventional weight mapping and 88-fold with the weight-expansion technique, owing to the increased digital preprocessing) makes the analog-only and projected full-system energy efficiencies similar (Fig. 6c; 7.09 TOPS/W and 6.94 TOPS/W using conventional weight mapping). With weight expansion, an analog-AI system using the chips reported in this paper could achieve 546.6 samples per second per watt (6.704 TOPS/W) at 3.57 W, a 14-fold improvement over the best energy-efficiency submitted to MLPerf (Fig. 6f), at 9.258% WER.

## Conclusions

In this paper we demonstrate the implementation of industry-relevant inference applications on analog-AI chips, specifically for speech recognition and transcription within the domain of NLP. We used a 14-nm analog inference chip to demonstrate SW_eq_ end-to-end KWS on the Google Speech dataset using a fully analog set-up and a novel AB technique. We then targeted the MLPerf RNNT on Librispeech, a data-center model with more than 45 million weights, mapped on more than 140 million PCM devices distributed over 5 different chip modules. By using a new weight-expansion method, we demonstrated a WER of 9.258% with an on-chip sustained performance that varies with tile usage, reaching a maximum of 12.4 TOPS/W and delivering an estimated system sustained performance of 6.7 TOPS/W.

These are, to our knowledge, the first demonstrations of commercially relevant accuracy levels on a commercially relevant model combining more than 140 analog-AI tiles, with neural-network activations being moved between those tiles with efficiency and massive parallelism. Our work indicates that, when combined with time-, area- and energy-efficient implementation of the on-chip auxiliary compute^20^, the high energy efficiency and throughput delivered during matrix–vector multiplication on individual analog-AI tiles can be extended to an entire analog-AI system, offering excellent sustained energy efficiency and throughput.

## Methods

### Chip fabrication and testing

**Extended Data Fig. 1:**
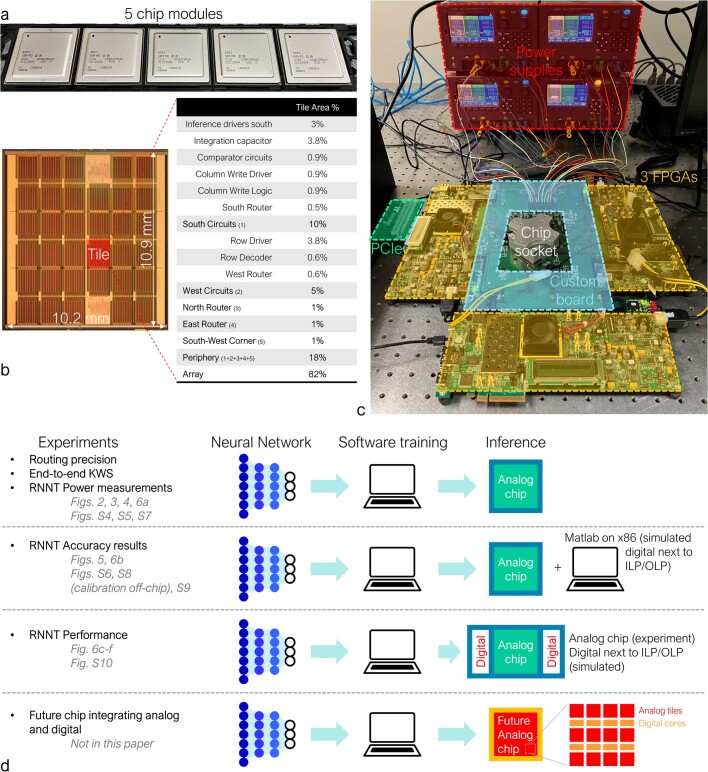
Analog in-memory computing chip and test platform. (a) Packaged modules used in the experiments. (b) Micrograph of the chip at H3 metal level, showing all analog tiles and the corresponding area breakdown. (c) The test platform is based on a chip socket mounted on a custom board driven by 3 Xilinx Virtex-7 VC707 FPGAs. Power is supplied by four Keysight E36312A. The three FPGAs are connected through the board, and the overall system is controlled by an x86 machine using a PCIe connector. (d) For routing precision, end-to-end KWS, and RNNT power measurements, the chip is run without any need to send intermediate data back to an x86 machine during the experiment. KWS models are trained for chip deployment using HWA methods. For RNNT accuracy measurements, intermediate data moves between the chip and an x86 machine. To predict the RNNT performance, we assume digital circuitry would be integrated on the chip next to the ILP/OLP pair, leading to predictions that should be very close to a fully integrated chip in which both analog tiles and digital compute cores are tightly integrated^20^.

Our experimental results were measured on chips built from 300-mm wafers with a 14-nm complementary metal-oxide-semiconductor front end, fabricated at an external foundry. PCM devices were added in the ‘back-end-of-line’ at the IBM Albany NanoTech Center. Mushroom-cell PCM devices were built with a ring heater with a diameter of approximately 35 nm and a height of around 50 nm (Fig. 1e) as the bottom electrode, a doped Ge_2_Sb_2_Te_5_ layer and a top electrode. Wafer characterization before packaging was performed on both 1-resistor macros and 1,024 × 2,048 array diagnostic monitors with on-chip sense amplifiers. After selection of high-yield dies, the wafer was diced and packaged into testable modules at IBM Bromont, as shown in Extended Data Fig. 1a,b.

Experiments were run by mounting the module on a socket connected to a custom-designed board driven by three Xilinx Virtex-7 VC707 field-programmable gate arrays (FPGAs) (Extended Data Fig. 1c). Four Keysight E36312A power supplies were used to power up the boards and the chip. In addition to the 1.5 V, 0.8 V and 1.8 V supplies mentioned in the main text, a 3.0 V power supply was provided but only during PCM device programming (not during inference). Finally, a supply of 0.75 V precharged the peripheral capacitors and set the lower limit for the on-chip digital-to-analog converters (DACs) used in PCM programming, and 0.3 V set the PCM read voltage and the ramp start voltage. These supplies were measured and reported in Fig. 6a as ‘Other’ voltage supplies. The three FPGAs were connected through the custom board and controlled by an x86 machine with a Peripheral Component Interconnect Express connector. All experiments were run using Xilinx MicroBlaze Soft Processor code and x86 MATLAB software wrapper (Extended Data Fig. 1c).

The off-chip combined transfer bandwidth on our chip is 38.4 Gbps, with a total of 384 input–output pins capable of operating at 100 MHz. Extended Data Fig. 1d shows that routing precision, KWS and RNNT power measurements were run without any additional intermediate data being sent back to the x86 machine. The RNNT accuracy results used the x86 for vector–vector operations and tile calibration. To model such digital operations in terms of performance, we simulated a digital circuitry just outside the ILP–OLP, based on a foundry 14-nm process design kit to implement optimized digital pipelines, control logic and registers. A future chip will eventually include the digital circuitry close to the analog tiles^20^.

### On-chip data conversion, analog periphery and 2D mesh routing

**Extended Data Fig. 2:**
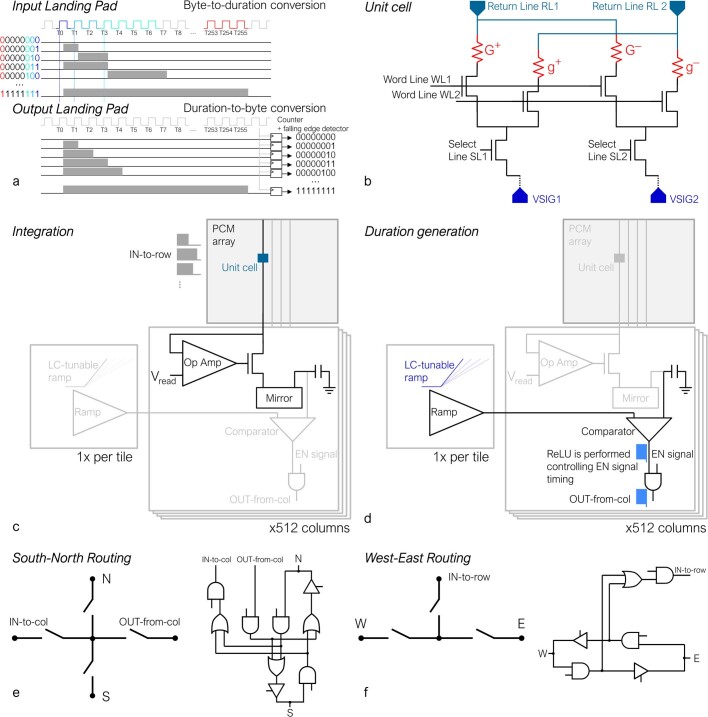
Analog circuits and routing configurations. (a) Input Landing Pad circuitry receives an 8-bit word, which is converted to a duration using a counter. Output Landing Pad circuitry receives one duration and converts it into an 8-bit word using 512 counters plus falling edge detectors. (b) Each tile has 512x512 unit cells, each consists of 4 PCM devices controlled by Word Lines and Select Lines driven by the tile West circuitry. During weight programming, Return Lines (RL) are driven at higher voltage (max 3V) and VSIG are grounded to enable PCM write. (c) During inference, in the integration phase, RL are grounded and one or both VSIG are biased at read voltage V_*read*_ = 0.3 V using a per-column operational amplifier. Current is then mirrored into a per-column capacitor. (d) For duration generation phase, capacitor voltage is compared against a shared and tunable ramp-voltage. The output of the comparator is a pulse with duration proportional to the voltage on the capacitor, gated by an EN signal using an AND gate. By properly tuning the timing of this EN signal with the Local Controller, activation functions such as ReLU or hard sigmoid can be implemented. (e) 512 durations are then sent into the parallel 2D mesh using a per-column South-North routing circuit. (This same circuit is also used to send pulses onto each column for PCM programming purposes). (f) Finally, West-East Routing enables durations to access another tile and/or to simply move across the tile. Since each borderguard can independently block or pass signals, complex routing patterns including multi-cast and even concatenation are supported.

Inputs were encoded as 8-bit digital words stored on an SRAM within each ILP. Conversion of 512 such digital words to 512 PWM durations was performed using clock-driven counter circuitry within each ILP. Data were then retrieved from the chip using the OLP, which internally performed the conversion from time to digital using 512 counters plus falling-edge detectors (Extended Data Fig. 2a).

Each analog tile consists of 512 × 512 unit cells (Extended Data Fig. 2b), each containing four PCM devices. Circuitry can implement a significance factor *F* > 1 but we adopted *F* = 1, meaning that *G*^+/−^ and *g*^+/−^ are the same, apart from intrinsic stochasticity. This enabled the implementation of 2-PCM-per-weight and AB methods, both requiring equal contribution from *W*_P1_ and *W*_P2_. Word lines and select lines were controlled by the west circuitry, selecting whether two or four PCM devices were connected to the edge capacitor. During weight programming, signals VSIG1 and 2 were kept at ground. Only one of the four PCM devices was programmed each time, by selecting the word, select and return lines. Weight programming was done in an iterative row-wise fashion^4^. During inference, VSIG1 and 2 were biased at a read voltage, *V*_read_, of 0.3 V, while signals RL1 and 2 were at ground.

Inference was achieved in two steps (Extended Data Fig. 2c). During the integration phase, PWM pulses activated in each row for a time proportional to the desired input magnitude (unlike ref. ^32^, these durations were not converted to analog voltages using DACs). *V*_read_ was forced by a per-column operational amplifier, which biased the entire bit line. These pulses were buffered along the row to maintain pulse-width integrity. Although IR drops did occur along columns, the wide wires stopped them being critical to degradation of MAC accuracy, especially when compared with other more-important factors such as peripheral circuit linearity and saturation effects. Current was then mirrored into a per-column capacitor, which could be tuned by the LC by connecting up to 8 parallel metal-oxide-semiconductor capacitors, where each capacitor was 50 fF (we typically chose 250 fF). The choices of capacitor size and range of tunability were based on the available column area, the expected current in the array, the integration time and the mirror ratios achievable. The summation over an entire 512-row tile was performed fully in analog, without the need for partial summation in the digital domain. In the wide-input case involving two vertically neighbouring tiles (Fig. 1i), summation over 1,024 rows (or even 2,048 in the two 2-PCM-per-weight case) was still fully performed in the analog domain, without any intermediate digitization. For layers that used wide input, the read operation during closed-loop tuning used this combined configuration, allowing an individual weight to experience and correct for the same non-idealities that it would experience in the eventual inference MAC. This provided significant mitigation from additional MAC error induced by combining tiles. Depending on the sign of the input, the current could be steered to either charge or discharge the capacitor. After current integration, the tile was disconnected and the output duration was generated. During this step, a tunable ramp circuit, shared among all columns, set a linear voltage ramp that was compared with the voltage on the 512 peripheral capacitors (Extended Data Fig. 2d). For each column, the output voltage started high, and when the comparator switched, the output duration ended, determining the duration of that particular output pulse, which is similar to the approaches in refs. ^33,34^. Finally, an AND port enabled or disabled the pulse output. With proper enable signal timings controlled from the LC, activation functions such as ReLU or hard sigmoid could be implemented on chip. The 512 durations were produced in parallel, exiting the tile on 512 individual wires. Area-efficient design choices (such as the use of a common ramp generator circuit shared across all the columns, the elimination of a conventional ADC and associated digital registers, as well as optimized full-custom layouts) enabled dedicated per-column circuitry at pitch, without the need for column multiplexers.

These generated durations left the tile and propagated towards the next tiles or the OLPs using the OUT-from-col path in Extended Data Fig. 2e. Per-column south–north routing circuitry allowed for full parallel duration processing, enabling either N–S or S–N connection (without entering the corresponding tile), collecting durations from the tile (OUT-from-col) or sending durations into the tile columns (IN-to-col) as used during weight programming^4^. Per-row west–east routing blocks enabled W–E or E–W duration propagation and IN-to-row communication, allowing durations to reach the rows inside an analog tile and/or to move across the tile to implement multi-casting (Extended Data Fig. 2f).

### Local Controllers

A user-configurable LC on each tile (Fig. 2a) retrieved instructions from a local SRAM. Each very wide instruction word (128 bits) included a few mode bits, as well as the wait duration (in cycles of around 1 ns given the approximately 1-GHz local clock) before retrieving a next instruction. Although some mode-bit configurations allowed JUMP and LOOP statements, most specified which bank of tile control signals to drive. Most of the 128 bits thus represent the next state of the given subset of tile control signals. This approach allowed for highly flexible tests and simplified design verification, with a small area penalty compared with predefined-state machines.

For example, the LC could configure 2D mesh routing to enable input access to analog tiles through the west circuitry (Fig. 2b) and MAC integration on the peripheral capacitors. The LC then configured the ramp and comparator used to convert the voltage on the capacitor into a PWM duration, avoiding energy-expensive ADCs at the tile periphery. Finally, the LC decided which direction (north, south, west or east) to send the generated durations, configuring the south 2D routing circuits^4,33^.

The LC also configured the ‘borderguard’ circuits at the four edges of each tile to enable various routing patterns. For example, Fig. 2c shows how durations from odd columns in the top tile could be merged together with durations from even columns from the bottom tile. This configuration was used on the RNNT Dec chip (Extended Data Fig. 7c).

### Measurement of reliable transmission of duration vectors

Inputs were transformed into durations in the ILP circuitry. Durations spanned between 0 and 255 ns, encoded using 8-bit words. To verify the reliability of these communication paths across the entire chip (Fig. 2d), we repeatedly multi-cast 512 input PWM durations from the southwest ILP to all six OLPs at the same time. These durations were uniformly randomly distributed between 0 and 50 ns at 1 ns granularity (1 GHz clock), and CDFs of the error between measured and transmitted duration across 2,048 vectors (1 million samples) are shown in Fig. 2d. This experiment was repeated for distributions spanning from 0 to 100, 150, 200 and 250 ns. The maximum error never exceeded 5 ns, with shorter durations exhibiting even smaller worst-case error (±3 ns), showing that durations can be accurately communicated across the chip. Although in this case errors were introduced by the double ILP–OLP conversion and unusually long paths, during conventional inference tasks, the MAC error was always dominated by the analog MAC.

### KWS network training, pruning and calibration

KWS is used in a wide variety of devices, such as personal and home assistants, to perform actions only after specific audio keywords are spoken. Latency and accuracy are important attributes. When used in an ‘always-ON’ configuration, raw power is also an advantage. When gated by a much simpler two-class front end that can detect audio input of potential relevance and wake up the multi-class KWS system, energy per task becomes the relevant figure of merit.

**Extended Data Fig. 3:**
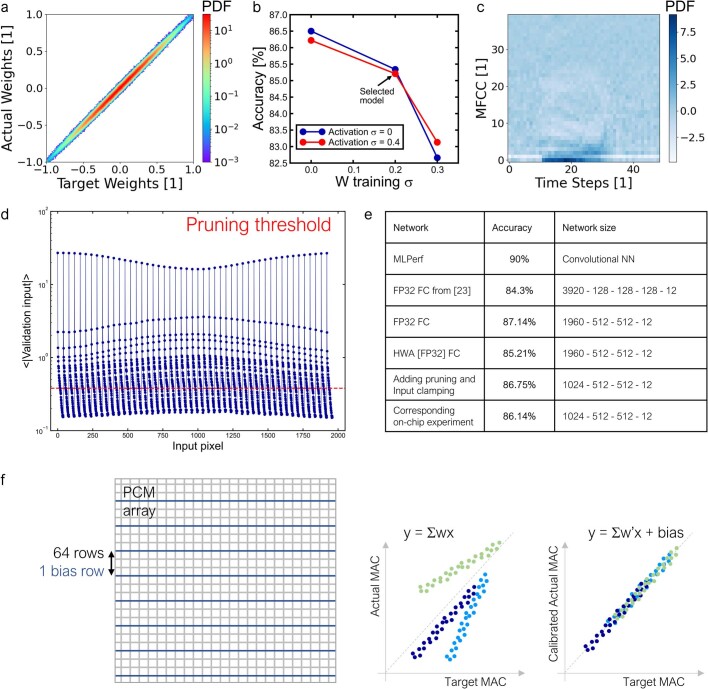
Keyword Spotting (KWS) Pre-processing and calibration. Hardware-aware (HWA) training was applied to improve model robustness against hardware imperfections, primarily due to programming errors. (a) Weight programming correlation for the HWA model. (b) Dependence of model accuracy on injected noise on weights and intermediate activations during training. (c) Mel-frequency cepstral coefficients (MFCC), representing the fingerprint for each keyword, are flattened and truncated before input to the fully-connected network. (d) The full input would require 1960 rows, however, to reduce the model to 1024-inputs, pruning is performed by removing all the inputs exhibiting a mean absolute validation input value lower than the indicated threshold. (e) The table shows a comparison of the KWS models and accuracies. (f) Since KWS is fully end-to-end on-chip, an on-chip calibration process is performed at the tile, leveraging 8 additional PCM bias rows to shift the MAC up/down to compensate for any intrinsic column-wise offsets. Slope of the MACs is compensated by re-scaling weights per-column. Calibration is performed using validation input data; inference results are reported for the test dataset.

The KWS network was trained using HWA techniques to make the network more resilient to analog memory noise and circuit-based non-idealities. We trained unitless weights on the interval (−1, 1) using weight clipping. In addition, we added normally distributed noise to these weights during each training mini-batch with a standard deviation of 0.02 (Extended Data Fig. 3a). We also added similarly distributed random noise with a standard deviation of 0.04 to output activations to mimic the imperfections expected from layer-to-layer activation transmission. We find that this simple noise model fits our analog system well and provides effective HWA training. We performed an extensive hyper-parameter search and picked a base learning rate of 0.0005 with a batch size of 250 for training. We found that including bias parameters for this network offered little benefit and therefore eliminated them from the model. We used adaptive moment estimation as the optimizer along with a weight decay (that is, L2 regularization) of zero. Finally, we used cross-entropy loss as our loss metric. The dependence of HWA accuracy for injected noise on weights and activations during training is shown in Extended Data Fig. 3b.

The KWS network performed several preprocessing steps before feeding the data into the FC layers. Input data (keywords) represented 1-second-interval voice recordings encoded as .wav files at a 16-kHz sampling rate. We computed the audio spectrogram, which is a standard way of representing audio information using the squared magnitudes of fast Fourier transforms taken at multiple time steps, using a window size of 30 ms and a stride of 20 ms. We then computed the Mel-frequency cepstral coefficients (MFCCs), which are a commonly used nonlinear transformation that accurately approximates the human perception of sound. We used 40 cepstral coefficients or bins per time slice. We also clipped the MFCCs to the range (−30, 30) to avoid any potential activation-rescaling problems going into our HW. This preprocessing resulted in a two-dimensional MFCC fingerprint for each keyword with dimensions of 49 × 40 (Extended Data Fig. 3c), and this is then flattened to give a 1,960-input vector. We also randomly shifted keywords by 100 ms and introduced background noise into 80% (the majority) of the training samples to make keyword detection more realistic and resilient.

To reduce the input size further and fit a 1,024-input-wide layer, we pruned the input data on the basis of the average of the absolute values of the validation input (Extended Data Fig. 3d). Pixels with average input intensity below a certain threshold were pruned, reducing the overall size to 1,024. Interestingly, pruning led to an accuracy improvement, as shown in the summary table in Extended Data Fig. 3e. Although our analog tiles can compute MAC on up to 2,048-element-wide input vectors, the AB method inherently uses both *W*_P1_ and *W*_P2_. Thus the maximum input size over which fully analog summation can be supported is reduced to 1,024.

Because the KWS network is fully on-chip, tile calibration needed to be performed in HW. A per-column slope and offset correction procedure was achieved in three steps. Weights were first programmed using the nominal target values. Next, 1,000 inputs taken from the validation dataset were used as input and the single-tile MAC results were collected to calculate the column-by-column slope scaling factors to be applied to the target weights. The tiles were then reprogrammed with the scaled weights. Finally, experimental MAC was shifted up or down by programming eight additional PCM bias rows available on each tile (Extended Data Fig. 3f). After tile calibration, the ReLU activation function was tuned using the same validation input and comparing the experimental result on validation data with the expected SW ReLU. The inference experiment was then performed on the test dataset. The calibration enabled compensation of column-to-column process variations and input-times-weight column dependencies (such as activation sparsity and residual weight leakage). As shown in the drift results on RNNT, tile weights typically showed good resilience to drift owing to the averaging effect. Bias weights required more-frequent updates, on the scale of days, to compensate for column drift, but this involved merely running a small inference workload and reprogramming the bias weights. Eventually, the tile weights also need to be re-programmed. Although we have not explored temperature-dependent conditions, we believe that the levels of PCM drift exhibited here would be sufficient to allow operation for a few days or even weeks, which is sufficient to keep model reprogramming for the purposes of PCM drift indistinguishable from model refresh for other purposes (such as resource balancing and model updates).

### RNNT weights and network mapping

**Extended Data Fig. 4:**
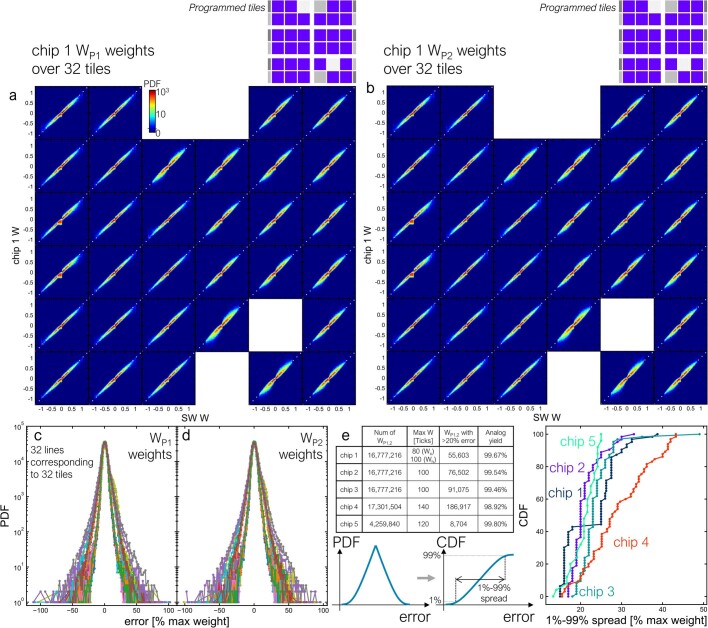
Experimental MLPerf RNNT weight programming. Experimental correlation between target and programmed weights on chip-1 over 32 tiles for both (a) W_*P*1_ and (b) W_*P*2_. (c),(d) The corresponding probability distribution functions (PDF) of errors, expressed as percentage of the maximum weight, reveal high-yield chips with very few erroneous weights. (e) Table showing the analog yield, or the fraction of weights with programming error within 20% of the maximum weight. After integration of the PDFs in (c,d), the corresponding cumulative distribution functions (CDFs) are computed and the 1%-99% spread is collected, providing 2 data points (one for W_*P*1_ and one for W_*P*2_) for each tile. The plot shows the corresponding CDFs for each of the five chips used in RNNT experiments. To control the peripheral circuitry saturation, some tiles have weights mapped into a smaller conductance range (Max W equal to 80), leading to a different 1%-99% spread, e.g. the points with increased spread on chip-1 CDF in (e).

To encode the MLPerf RNNT weights, we used five chips. Iterative weight programming enabled accurate tuning of the conductances to match the target weights. Heat maps correlating the target and the measured chip-1 weights on each of the 32 tiles are shown for *W*_P1_ and *W*_P2_ in Extended Data Fig. 4a,b. The corresponding error for each tile, expressed as the fraction of the maximum weight, is shown in Extended Data Fig. 4c,d for *W*_P1_ and *W*_P2_. To compare the weight programming in the five chips used for the RNNT experiment, we calculated the CDF on the basis of the data shown in Extended Data Fig. 4c,d and extracted the spread between 1% and 99%. In this way, two data points were extracted for each tile, one for *W*_P1_ and one for *W*_P2_. The chip analog yield, measured as the fraction of weights with a programming error of less than 20% of the maximum weight magnitude, is around 99% (Extended Data Fig. 4e). Chip 4 has a slightly lower yield because the corresponding maximum *W*, defined as the coefficient used to rescale weights from MLPerf (around [−1, 1]) to integers, is larger because more signal was required, causing greater weight saturation. Extended Data Fig. 4e shows the spread distributions for each of the five chips.

**Extended Data Fig. 5:**
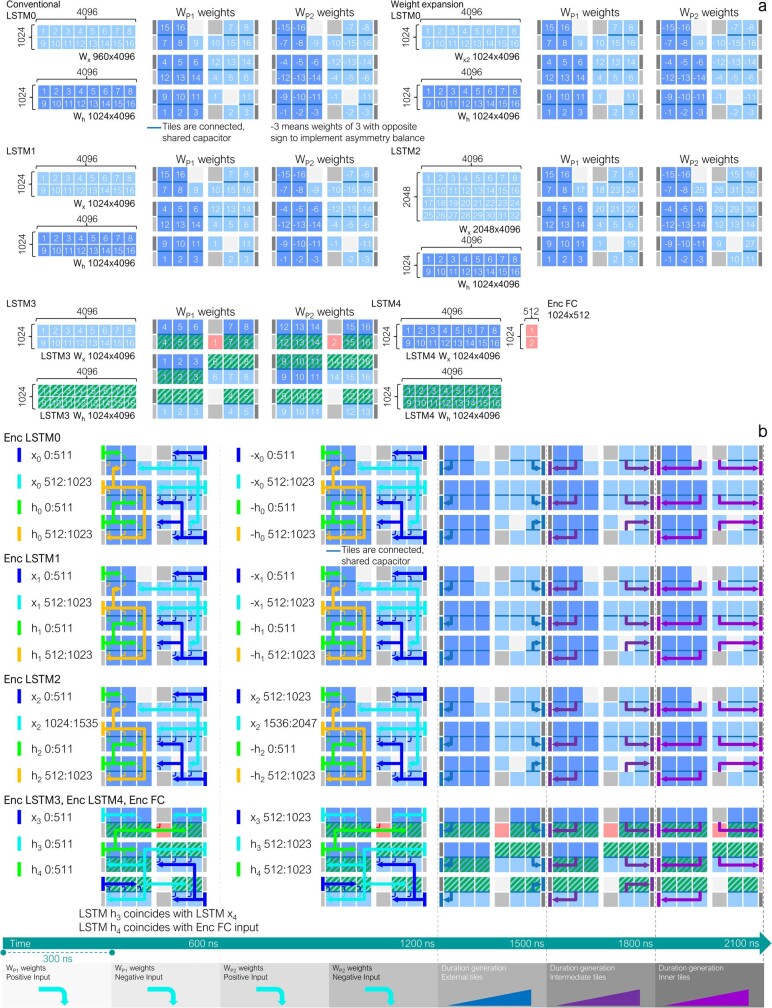
MLPerf RNNT Encoder mapping and signal routing on chip. (a) RNNT Encoder LSTMs weights are represented by two large matrices, W_*x*_ which multiplies the LSTM input, *x*, and W_*h*_ which multiplies the LSTM recurrent signal, *h*. Depending on the sizes of *x* and *h*, a variable number of tiles is required. Both conventional and weight expansion mappings are shown for Enc-LSTM0. In addition, Enc-LSTM0, Enc-LSTM1 and the W_*h*_ of Enc-LSTM2 implement Asymmetry Balance. Tiles connected with a dark blue line have shared capacitors, enabling 2048-wide analog MAC. In Enc-LSTM0, Enc-LSTM1 and Enc-LSTM2, MACs from tiles 1,9 and 2,10 are summed in digital, while all the other pairs are summed on-chip in analog. (b) Every analog MAC on the encoder chips requires seven 300 ns time steps to process, including digitization of the output. During the first four steps, MAC operations are performed, providing input signals from ILPs as indicated in the figure. In cases where AB is used (Enc LSTM0, Enc LSTM1, W_*h*_ portion of Enc LSTM2), opposite-signed inputs are provided in two of the four MAC time steps. During the last three time steps, MAC results are sent out to the OLPs.

The RNNT encoder weights were mapped using the first four chips, as shown in Extended Data Fig. 5a. The large *W*_*x*_ and *W*_*h*_ matrices used for encoder LSTMs all show a size of 1,024 × 4,096 except for the conventional Enc-LSTM0 (*W*_*x*_ is 960 × 4,096) and Enc-LSTM2 (*W*_*x*_ is 2,048 × 4,096). Enc-LSTM0, Enc-LSTM1 and the *W*_*h*_ matrix of Enc-LSTM2 implement AB. In Enc-LSTM0, Enc-LSTM1 and Enc-LSTM2, summation of *W*_*x*_ and *W*_*h*_ MACs was performed off-chip at the x86 host, whereas chip 4, implementing Enc-LSTM3 and Enc-LSTM4, performed this entire summation on-chip in analog. Furthermore, blocks 1(−1), 9(−9) and 2(−2), 10(−10) of Enc-LSTM0 W_**x**_ and Enc-LSTM1 *W*_**x**_, and blocks 1(9), 17(25) (*W*_P1_(*W*_P2_)) and 2(10), 18(26) were summed in digital after on-chip analog MAC. Finally, Enc-FC was implemented on chip 4. Any spot where tiles were connected by sharing the peripheral capacitor in the analog domain (Fig. 1i) is highlighted with a dark-blue bar. We did not map biases in analog memory but instead incorporated them in the already existing off-chip digital compute, by combining them into the calibration offset with no additional cost. Thus these biases were always applied with FP32 precision. No network retraining was applied.

To provide input data and collect MAC results in a massively parallel fashion from or to the ILPs–OLPs, complex routing paths were programmed, leveraging the flexibility of the LCs (Extended Data Fig. 5b). In the RNNT encoder, after each MAC, the data needed to go through input–output for off-chip digital processing. Each full operation (including input, MAC, duration generation and output digitization) took 2.1 μs. The input arrows show multi-cast in parallel to one or more analog tiles with MAC operations occurring on those tiles. Output MACs were provided to the OLPs in three time steps owing to the small number of OLPs.

**Extended Data Fig. 6:**
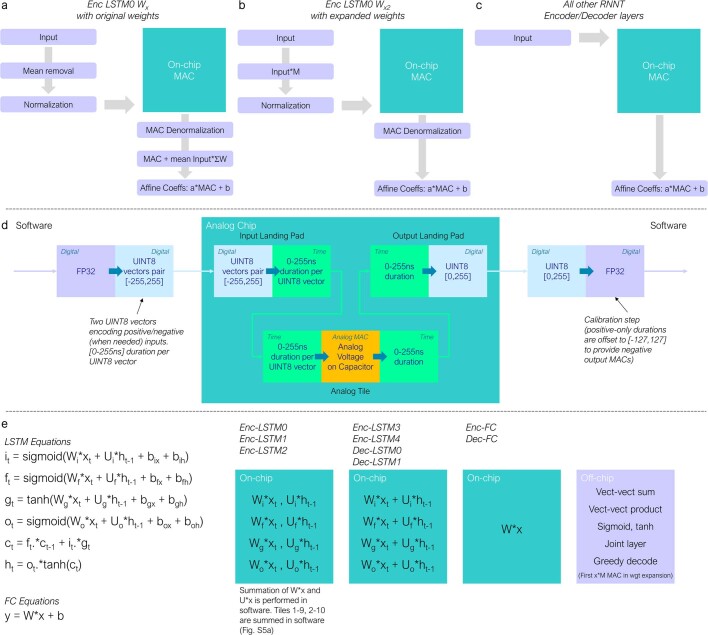
MLPerf RNNT Enc-LSTM0 input processing. Due to the large sensitivity of the first Encoder LSTM W_*x*_ matrix, input data is digitally pre-processed to increase the MAC signal-to-noise ratio. (a) Using the original MLPerf weights, input data is first shifted to zero-mean and normalized to a common maximum input amplitude. Then, on-chip MAC is performed, followed by MAC digital denormalization and addition of mean Input*ΣW. This last operation collapses to a simple single number (mean Input) times one 4096-vector represented by the column-wise sum of weights, not requiring a full digital 1024*4096 MAC operation. Only after these steps the affine coefficients (slope and offset correction) are applied. (b) When using expanded weights, the mean removal is no longer needed since the random matrix M introduced in the main paper has zero mean, leading to naturally near zero-mean input values, thus simplifying input pre-processing. (c) All the other RNNT blocks (rest of Encoder and Decoder) only apply affine coefficients, without any normalization or mean removal. (d) Data-type conversion during the MLPerf RNNT inference: software computation is performed in FP32. Before access to the chip, data is converted into a pair of UINT8 vectors, one for each polarity of the incoming activations. This data is loaded on-chip in the ILP, then converted into time duration and used as MAC input (‘negative’ inputs are durations sent during the second and fourth integration steps, as shown in Extended Data Figs. 5b and 7d). MAC output is represented as analog voltage on a capacitor, then converted into time duration by the peripheral circuitry (details in Extended Data Fig. 2d). Finally, the OLP converts durations into UINT8. Data is sent off-chip and converted in FP32 during the calibration phase. (e) Overall view of the equations solved in the RNNT: essentially, all MACs are performed on chip, while all vector-vector operations, non-linear activations and biases are computed in software. The joint layer is implemented in software, as explained in Fig. 4.

RNNT experiments implemented MAC on-chip, whereas tile affine calibration (shift and scale) and LSTM vector–vector computations were performed in SW (MATLAB SW running on x86). In particular, the first Enc-LSTM0 *W*_**x**_ required careful input-signal management to maximize the signal-to-noise ratio, owing to the large sensitivity of the WER to any noise on its weights. Extended Data Fig. 6a shows that, in the case of Enc-LSTM0 *W*_**x**_, the input data, which naturally exhibits a wide dynamic range, was first shifted to zero-mean, followed by normalization to maximum input amplitude. The preprocessed input was then used for analog MAC. The MAC results were later denormalized back in SW, where the input mean contribution was added (which collapses to the product of one number, the mean value of the input image, and one vector, the sum of weights for every column) and the affine coefficients for calibration were applied.

In the case of expanded weights (Extended Data Fig. 6b), the input first underwent MAC with the random matrix *M* (such a matrix has random normal weights but is fixed across all inputs). Because the product of an input with a matrix with zero mean value generates an output with near-zero mean value, there was no need to apply the zero-mean shift, although normalization to maximum amplitude was still performed. After the analog on-chip MAC, the results are denormalized and the usual calibration was applied. For every other layer (Extended Data Fig. 6c) in the RNNT, the inputs were used directly as tile activations and the MAC was calibrated with the usual affine coefficients. All affine coefficients are calculated by comparing experimental and expected SW MAC using 2,000 input frames from the training dataset for each Enc–Dec layer. Data were linearly fitted to obtain the slope and offset coefficients.

**Extended Data Fig. 7:**
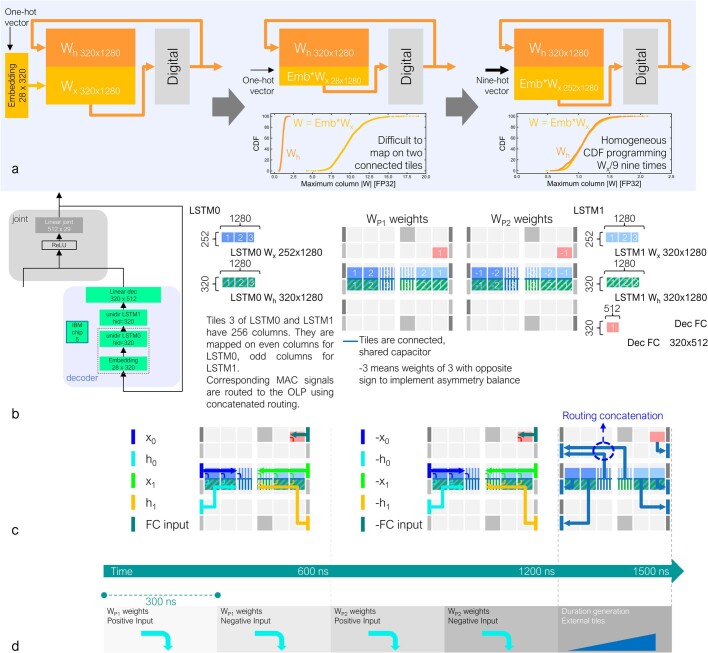
MLPerf RNNT Decoder mapping and signal routing on chip. Instead of processing Embedding Emb and Dec-LSTM0 W_*x*_ layers separately, we first compress in one single matrix the product Emb * W_*x*_. At this point, the first Dec-LSTM0 shows Emb * W_*x*_ matrix with a (28*1280) size, contrasting with the (320*1280) W_*h*_ size to sum directly in analog. To balance signal magnitude, nine copies of Emb * (W_*x*_/9) are programmed, achieving comparable weight absolute magnitudes. (b) Weight mapping and (c) signal routing implementing Asymmetry Balance. Routing from tiles to Output Landing Pads utilizes implicit vector concatenation on the 2D mesh, enabling more efficient data transport. (d) The processing of one full frame requires 5 time steps of 300 ns each.

Extended Data Fig. 6d shows a detailed description of all data-type conversions. All SW computations were performed in FP32. For transmission to the chip, data were converted into INT9 (UINT8 plus sign) and UINT8 vectors were loaded into the ILP. Here, durations were generated and sent to the tiles where the analog MAC was performed, collecting an analog voltage on a peripheral capacitor. Once the UINT8 vectors were loaded into the ILP, ‘negative’ durations were sent during integration of the second or fourth time step, as shown in Extended Data Figs. 5b and 7d. Finally, charge integrated onto column-wise capacitors was converted by the peripheral circuitry into durations that were sent to other tiles or to the OLP, which converted them back into UINT8. Data were then sent off-chip and transformed back into FP32 during the calibration stage. Extended Data Fig. 6e shows a summary of the equations, highlighting that essentially all MACs were performed on-chip, whereas vector–vector, bias and nonlinear activations were computed in SW. The joint layer was in SW.

Extended Data Fig. 7 shows the details of Dec mapping and signal routing. To account for the Emb layer (Extended Data Fig. 7a), we first collapsed Emb and Dec-LSTM0 *W*_**x**_ layers into a single Emb × *W*_**x**_ matrix with size 28 × 1,280, which receives one-hot input vectors. This multiplication is perfectly equivalent in SW, but led to large weights in the Emb  ×  *W*_**x**_ matrix compared with *W*_*h*_, as shown in the first set of CDFs, reporting the maximum weight for each column. Because MAC results from Emb  ×  *W*_**x**_ and *W*_*h*_ are summed directly in the analog domain with a shared capacitor, weight values cannot be arbitrarily scaled. To overcome this problem, 9 copies of the 28 × 1,280 Emb × *W*_**x**_ matrix were programmed and the 28 inputs duplicated onto 9 × 28 rows, leading to a similar amount of signal with *W*_*h*_. This allowed us to effectively distribute these large weights over 9 unit cells, while ensuring that the analog summation will aggregate both the Emb  × *W*_**x**_ and the *W*_*h*_ contributions with the correct scaling.

Dec weight mapping used AB (Extended Data Fig. 7b) and signal routing enabled parallel input and output of all signals (Extended Data Fig. 7c). Here, routing concatenation was used to efficiently combine the signal from two different tiles into the same OLP. The full input–MAC–output processing time is 1.5 μs (Extended Data Fig. 7d).

Unlike the KWS experiment, the MLPerf repository mandates that inference be performed with the validation dataset. The RNNT MLPerf inference experiments shown in Fig. 5 were done by inputting the full validation dataset into the first chip, saving the output results on the x86 machine, swapping in the second chip and continuing the experiment, using the previously saved outputs as new inputs. This procedure was repeated for all five chips, ensuring a consistent example-by-example cascading, as in a fully integrated system. Mapping even-larger models, using a weight-stationary configuration, can be supported with improved memory density (including stacking of multiple layers of PCM in the back-end-of-line), multi-chip modules and even multi-module solutions, with careful neural-network partitioning to minimize inter-module communication that would be energy expensive.

### RNNT MAC and end-to-end accuracy

**Extended Data Fig. 8:**
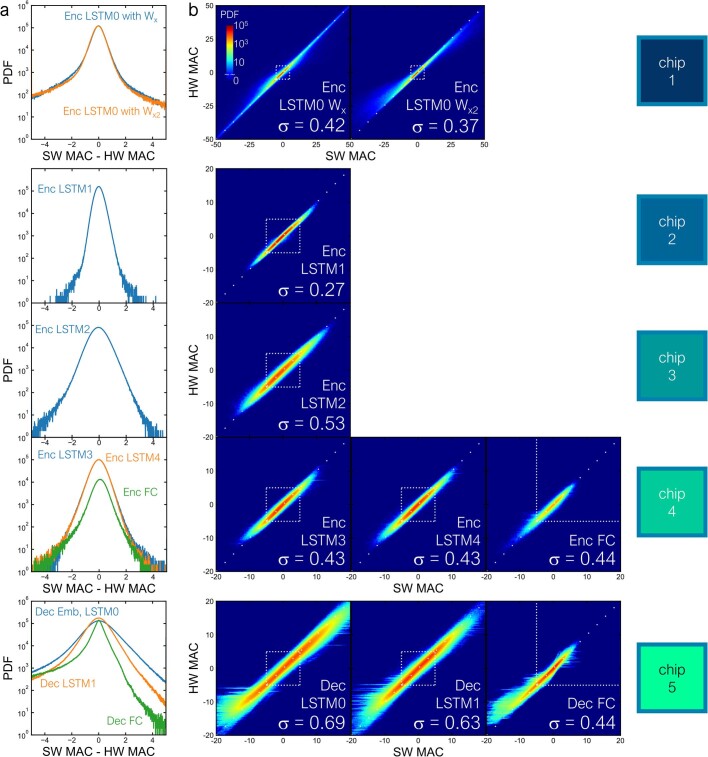
Experimental MLPerf RNNT MAC. (a) Error histogram and (b) correlation between experimental and target MAC are shown for every chip used during the RNNT inference experiment. On each correlation, white dotted lines highlight the main region of interest, since MACs are followed by sigmoid, tanh or ReLU which naturally filter out portions of MAC. The spread, *σ*, is calculated only within the highlighted Regions Of Interest (ROI). Data from both original Enc-LSTM0 and weight-expanded Enc-LSTM0 are reported, showing a better sigma for the weight expanded case. Enc-LSTM2, Enc-LSTM3, and Enc-LSTM4 show larger spread due to partial (Enc-LSTM2) or no (Enc-LSTM3, Enc-LSTM4) application of Asymmetry Balance. In addition, Enc-LSTM2 MAC is calculated on larger (3072 instead of 2048) inputs. Finally, decoder layers show larger *σ*, maybe caused by higher capacitor/Output Landing Pad saturation effects, which however have little impact on the overall WER, as revealed by the accuracy results in the main paper (Fig. 5a,b).

**Extended Data Fig. 9:**
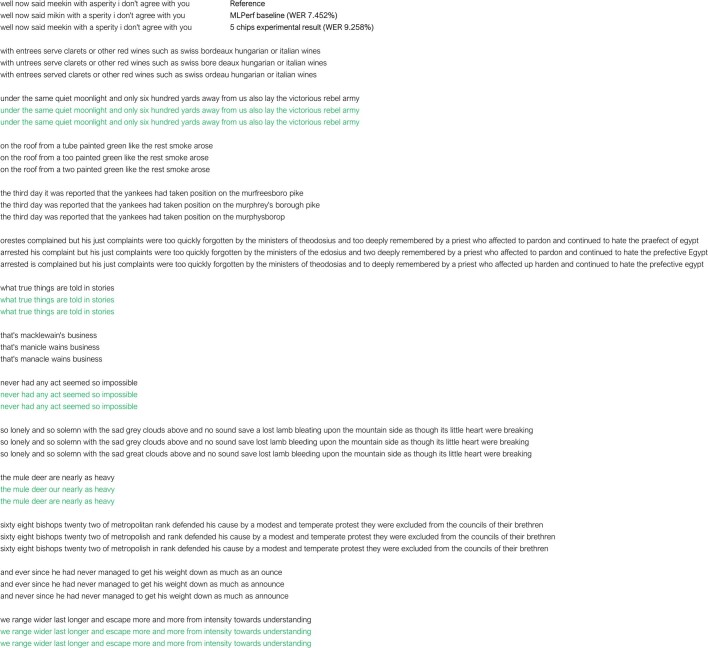
Librispeech transcribed sentences. First ten transcribed sentences from the Librispeech validation dataset. The first line shows the reference sentence, the second line corresponds to the MLPerf baseline prediction (WER 7.452%), and the third line shows the sentences produced by our five-chips experiment including the weight expansion technique (WER 9.258%).

Experimental MAC details are shown in Extended Data Fig. 8. The error distributions and MAC correlations are shown for every chip. In all figures, a dashed region highlights the main regions of interest for that MAC. For LSTM layers, the region of interest corresponds to the [−5, 5] range, because outside that range the ensuing sigmoid or tanh function can be expected to fully saturate (for example, the output will always be −1 or +1, being almost completely independent of any variations on the input). Similarly, the regions of interest for the FC layers are mostly the positive MACs because of the ReLU activation function. In this specific case, Enc-FC and Dec-FC are summed before ReLU, so slightly negative contributions could also matter. We plotted the regions of interest to be where MAC > −5. The reported standard deviation *σ* computes the error for SW MAC in [−5, 5] for LSTMs and [−5, inf] for FC layers. Comparison between the original *W*_**x**_ and the weight-expanded *W*_**x**2_ for Enc-LSTM0 is also provided. Extended Data Fig. 9 shows examples of transcribed sentence output from the experiments in Fig. 5 that show an almost iso-accuracy WER. Transcription results are in good agreement between the MLPerf RNNT model implemented in analog HW and in SW, indicating that the effective bit-precision of our HW demonstration is *n*_bits_ = 4.097 for 9.475% WER and *n*_bits_ = 4.153 for 9.258% WER (weight expansion), on the basis of comparison with the full network (no joint FC) curve in Fig. 4c.

### Performance simulation and power measurements

**Extended Data Fig. 10:**
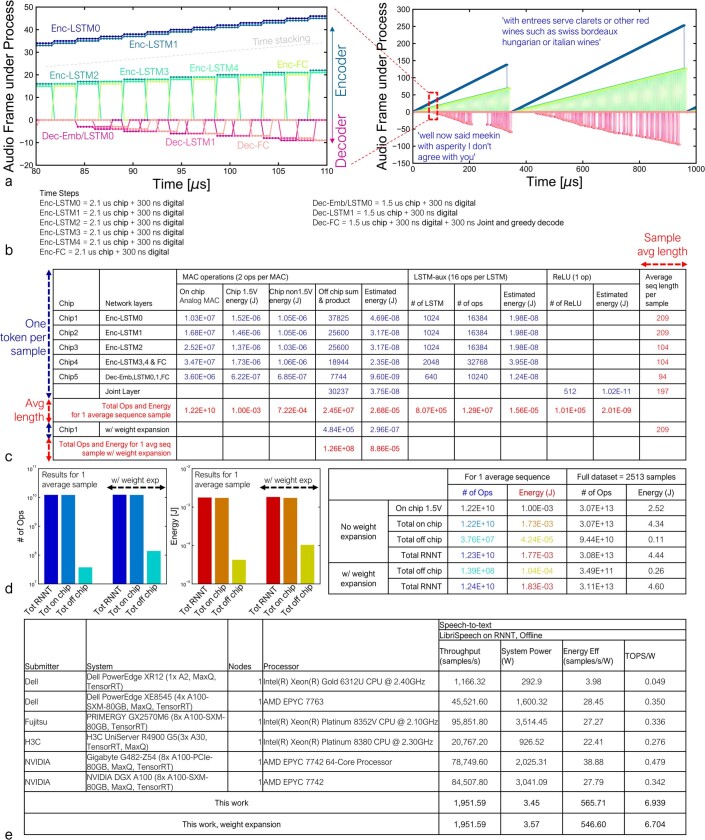
System performance estimation. To calculate processing time for RNNT on an integrated system as described in Fig. 6, (a) a simulator based on the MAC runtime on the actual chip and plausible digital processing is considered based on (b) specific timing assumptions stemming from our experiment and prior architectural work^20^. (c) Detailed breakdown of operations and energy across the 5 chips, including additional digital operations required to process activations from chips. (d) Total on-chip and off-chip number of operations and energy, including measured analog operations (this paper) or estimates for digital ops^20^. (e) Comparison with MLPerf submissions on RNNT shows a 14 × advantage in energy-efficiency.

The proposed 5-chip RNNT implementation is not integrated with digital processing, but we can estimate the time needed to process the entire dataset by combining the MAC processing times and energies from the analog chips with the estimated digital processing times and energies that we tabulated previously in our architecture paper^20^. Extended Data Fig. 10a shows a timing simulation describing the execution of RNNT layers for processing all 2,513 input audio samples, accounting for all pipelining, time stacking, recurrence and Dec steps. We assume times of 2.1 μs and 1.5 μs for the Enc and Dec layers, respectively, which includes all duration generation, and a relatively conservative 300 ns for the digital processing of each layer. Given these assumptions, the entire dataset can be evaluated in 1.2877 s, corresponding to a rate of 1,951.59 samples per second. Combined with the power measurements below, these numbers can be used to extrapolate the analog-AI RNNT system performance.

Power measurements for RNNT were done using a set of 32 exemplar input vectors that filled up the ILP SRAM to capacity. By overflowing the address pointer of the ILP, it is possible to repeat the same set of 32 vectors ad infinitum. Together with JUMP instructions in the LCs resetting the program counters to the start of program execution, this allowed a real-time current measurement from the voltage supplies for the inference tasks. In these measurements, all 7 (or 5) phases of the Enc (or Dec), including 4 integration phases and 3 (or 1 for the Dec) duration generation phases were included. This accounted not just for the MAC integration, but also for the subsequent cost of generating, transporting and digitizing the MAC results. The measured powers are shown in Fig. 6a.

Using the energy and execution-time models from our architecture study^20^, the total digital energy (for all the tasks performed off-chip in SW to support the experiments shown in this paper) is estimated to be 0.11 J for nominal Enc-LSTM0 and 0.26 J for weight-expansion Enc-LSTM0. The total number of digital operations and a detailed breakdown are shown in Extended Data Fig. 10c,d.

Although several compute-in-memory or near-memory approaches based on SRAMs and digital compute^35–38^ have been presented in the literature, most of these do not address the energy and time costs of reloading weights, thus making direct side-by-side comparisons against NVM-based weight-stationary approaches difficult. However, several NVM compute-in-memory studies have focused on the macro-level^32,34,39,40^^,^^41^, without accounting for data transport, control or chip infrastructure (such as clocking) costs. They are also usually at a much smaller scale (sometimes less than 1 million parameters^7^) than the work here, making a fair assessment of both the accuracy of large models and the associated sustained TOPS/W values difficult.

We have instead compared our sustained power and performance values against other reported system numbers for the same RNNT task from MLPerf, as shown in Extended Data Fig. 10e. By weighting the sustained power measurements for individual chips with their corresponding activity factors from the timing simulations shown in Extended Data Fig. 10a, the total system energy and corresponding aggregate TOPS/W values for our system are calculated to be 4.44 J and 6.94 TOPS/W, respectively (4.60 J and 6.70 TOPS/W for *W*_*x*__2_). Although our evaluations in Fig. 6 do not include some external components used in real systems, such as system buses and voltage regulators, this TOPS/W energy efficiency is still more than an order of magnitude better than the best published result for this task.

The relatively small number of digital operations in the network implies that considerable benefits may yet be obtained by improving the raw analog MAC energy efficiency (currently 20 TOPS/W). This could be enabled by shorter integration times, more-efficient analog opamps and/or lower-conductance devices. Instead, a substantial drop-off in energy efficiency, down to 12.4 TOPS/W for chip 4 (Fig. 6c), occurs as a result of the on-chip infrastructure, such as the landing pads, which need to be exercised at the end of each MAC. This highlights the need for on-chip digital compute cores, potentially in proximity to the same chip, and using the same local 2D mesh for data transport as described in our architecture study^20^.

MLPerf submissions for RNNT exhibit performance efficiencies ranging between 3.98 and 38.88 samples per second per watt, using system power that ranges from 300 to 3,500 W, assuming the use of large batches to maximize efficiency. Our work inherently assumes a mini-batch size of 1. Although we assume that additional samples are available to keep the pipeline full, our projections are effectively independent of mini-batch size. Under these conditions, an analog-AI system using the chips reported in this paper could achieve 546.6 samples per second per watt (6.704 TOPS/W) at 3.57 W, a 14-fold improvement over the best energy-efficiency results submitted to MLPerf. Reduction in the total integration time through precision reduction, hybrid PWM^40^ or bit-serial schemes can improve both throughput and energy-efficiency, but these could suffer from error amplification in higher-significance positions. Future efforts will need to address their impact on MAC accuracy for commercially relevant large DNNs.

## Online content

Any methods, additional references, Nature Portfolio reporting summaries, source data, extended data, supplementary information, acknowledgements, peer review information; details of author contributions and competing interests; and statements of data and code availability are available at 10.1038/s41586-023-06337-5.

## Data Availability

The MLPerf RNNT model is available from the MLPerf repository^8^.

## Code availability

The KWS weight-programming pseudo-code is available from Zenodo (https://zenodo.org/7992452).
